# mHealth Incentivized Adherence Plus Patient Navigation (MIAPP): protocol for a pilot randomized controlled trial to improve linkage and retention on buprenorphine for hospitalized patients with methamphetamine use and opioid use disorder

**DOI:** 10.1186/s13722-025-00538-1

**Published:** 2025-01-29

**Authors:** Elenore P. Bhatraju, Devin N. Kennedy, Alexander J. Gojic, Matthew Iles-Shih, Joseph O. Merrill, Jeffrey H. Samet, Kevin A. Hallgren, Judith I. Tsui

**Affiliations:** 1 Department of Medicine, Division of General Internal Medicine, University of Washington/Harborview Medical Center, 325 9Th Avenue, Box 359780, Seattle, WA 98104 USA; 2 Department of Psychiatry and Behavioral Sciences, University of Washington, Seattle, WA USA; 3 Clinical Addiction Research and Education (CARE) Unit, Section of General Internal Medicine, Department of Medicine, Boston University Chobanian & Avedisian School of Medicine and Boston Medical Center, Boston, MA USA

**Keywords:** Buprenorphine, Opioid related disorders, Mobile Health (mHealth), Video directly observed therapy, Methamphetamine use

## Abstract

**Background:**

Initiation of buprenorphine for treatment of opioid use disorder (OUD) in acute care settings improves access and outcomes, however patients who use methamphetamine are less likely to link to ongoing treatment. We describe the intervention and design from a pilot randomized controlled trial of an intervention to increase linkage to and retention in outpatient buprenorphine services for patients with OUD and methamphetamine use who initiate buprenorphine in the hospital.

**Methods:**

The study is a two-arm pilot randomized controlled trial (N = 40) comparing the mHealth Incentivized Adherence Plus Patient Navigation (MIAPP) intervention to treatment as usual. Development of the MIAPP intervention was guided by the information-motivation-behavioral skills model and combines financial rewards via mobile health-based adherence monitoring with the “human touch” of a patient navigator. Participants receive financial incentives for submitting videos of themselves taking buprenorphine via smartphone. The Patient Navigator reviews videos and provides treatment adherence coaching, care coordination and motivational enhancement. The intervention is introduced prior to hospital discharge and is offered for 30 days. The primary outcome is linkage to outpatient buprenorphine care within 30 days of hospital discharge. Secondary outcomes include retention on buprenorphine 90 days post discharge, hospital readmissions, and past 30-day methamphetamine use.

**Discussion:**

Interventions are needed to increase linkage and retention to outpatient buprenorphine among hospitalized patients with OUD, especially for people who co-use methamphetamine. We will examine the MIAPP intervention to improve buprenorphine adherence and linkage to outpatient treatment in a pilot randomized controlled trial which will provide valuable insights about research approaches for hospitalized patients with substance use disorder.

*Trial registration number*: NCT06027814. Date of Initial Release: 08/30/2023. Protocol Version: 03/21/2024.

**Supplementary Information:**

The online version contains supplementary material available at 10.1186/s13722-025-00538-1.

## Introduction

Opioid use disorder (OUD) has become recognized as a major public health crisis, with overdose deaths in the United States doubling from 2016 (42,249 opioid-related overdose deaths) to 2022 (> 84,000) [1, 2]. More recently, the OUD crisis has been further exacerbated by the widespread emergence of methamphetamine use concurrent with use of opioids—coined as the “fourth wave” of the overdose epidemic. A 2021 report issued by the Centers for Disease Control and Prevention using 2019–2020 data reported that 40% of overdose deaths involving illicitly manufactured fentanyl also involved stimulants [3]. Mitigating the OUD crisis requires health systems to deliver interventions that can effectively address both OUD and methamphetamine use.

Medications for OUD—including buprenorphine and methadone—are effective in reducing opioid use [4] and in lowering risk of fatal and non-fatal opioid overdoses, making them vital tools for combatting the OUD crisis [5, 6]. Buprenorphine has some benefits compared to methadone: a more favorable safety profile [7]; simpler logistics that do not require frequent visits to a federally licensed opioid treatment program for direct observation of dosing in contrast [8]. Multiple studies, however, demonstrate that among people with OUD, those who also use methamphetamine are less likely to initiate medications for OUD and less likely to be retained in treatment compared to those who do not use methamphetamine [9, 10].

Hospital settings are uniquely positioned to support patients with substance use disorders who are not explicitly seeking substance use treatment [11]. People who use opioids and stimulants are more likely to visit the emergency department [12], be admitted or readmitted to the hospital [13], and to leave against medical advice compared to those who use opioids alone [14]. This group also has increased risk for medical conditions that can require acute care (e.g., blood borne infections [15], complications from HIV and hepatitis C virus [16, 17]). For many patients, the experience of hospitalization can be a “reachable moment” where they move to an “action” stage of change with regard to initiating medications for OUD [18]. A growing number of hospitals have developed specialty teams and protocols to help initiate buprenorphine for patients with OUD during their hospitalization. Research shows that addiction consultation services that initiate buprenorphine during hospitalization with a plan to continue outpatient treatment can increase engagement in subsequent outpatient treatment [19].

For patients who are initiated on buprenorphine during a hospitalization, successful linkage to an outpatient buprenorphine provider after discharge is essential for continuing treatment and reducing longer-term OUD-related risk. However, linkage rates remain suboptimal: observational studies suggest that among patients initiated on buprenorphine in the hospital, only about 50% link to an outpatient buprenorphine provider [20]. Moreover, the likelihood of linking to outpatient buprenorphine treatment is 40% lower for people who use methamphetamine [21]. Discharge from the hospital can be a difficult transition. Specifically for buprenorphine, hospital discharge requires a transition to taking medication unsupervised and establishing care at an outpatient treatment setting to continue receiving the medication. Interventions are needed to support patients in continuing to take their buprenorphine after they are discharged from the hospital and to facilitate their linkage to an outpatient treatment setting.

In response to this need, we have developed the “mHealth Incentivized Adherence Plus Patient Navigation” (MIAPP) intervention to support patients with adhering to their buprenorphine medication and with linking to outpatient buprenorphine treatment services following discharge from the hospital. The intervention leverages the benefits of contingency management through financial incentives, mobile health (mHealth), and patient navigation with the goal of enhancing motivation for continuing treatment and supporting patients during the potentially difficult period of transitioning out of the hospital. The intervention includes a combination of evidence-based components. Patient navigators are trained, culturally sensitive healthcare workers who assist patients with complex medical and psychosocial conditions to overcome structural and individual-level barriers to care [22]. Patient Navigator interventions have successfully been used for management of numerous health conditions that overlap with OUD, including hepatitis C [23, 24] and HIV [25], and for persons with both HIV and substance use disorders [26]. Patient Navigators can provide multiple forms of support to hospitalized patients [27] including an important human connection that could complement mHealth by providing flexibility and personalization, the lack of which can be a significant barrier to engagement [28]. Contingency management, providing positive rewards for desired behaviors, has robust evidence in the treatment of methamphetamine use disorder [29]. The National Institute on Drug Abuse has now prioritized research on incentive-based interventions with a goal of increasing treatment reach, reproducibility, privacy and engagement, while decreasing cost [30]. Video-DOT (directly observed therapy) is a method of confirming medication ingestion using an asynchronous video platform via mobile phone application. Modeled after in-person directly observed therapy, video-DOT enables patients to be observed in their own environment without the challenges that can arise pursuing attendance at a clinic for in-person observation. Video-DOT has been successfully utilized for tuberculosis [31, 32] for over a decade and has been piloted in both opioid treatment program settings to monitor methadone dosing [33, 34] as well as outpatient buprenorphine clinics [35–37].

While contingency management, mHealth, and patient navigation have been studied individually, we are not aware of interventions that have combined these approaches to support retention in medications for OUD treatment, particularly for patients transitioning out of the hospital (a time in which many patients struggle to continue treatment after discharging). Here we describe the study design and protocol of a pilot randomized controlled trial of the novel MIAPP intervention tested in hospitalized patients with OUD who use methamphetamine initiating sublingual buprenorphine during hospitalization. The primary goal in conducting the pilot trial is to evaluate the feasibility and acceptability of the novel intervention and to inform the design of a future larger-scale trial.

### Motivation for pilot study

The investigative team believed that the novelty of the MIAPP intervention and the setting/timeframe in which it is offered to patients warranted investigation in the form of a pilot study to determine if the intervention and research procedures were feasible. Successful completion of a pilot randomized controlled trial would help demonstrate the capability of performing a larger, fully-powered randomized controlled trial that could more definitively test whether the MIAPP intervention was effective in supporting hospitalized patients continue buprenorphine treatment as they transition out of the hospital. Therefore, we designed the current pilot study to emulate the procedures that could be followed for a future fully-powered clinical trial, but with a smaller sample.

## Methods

### Study design

**Fig. 1:**
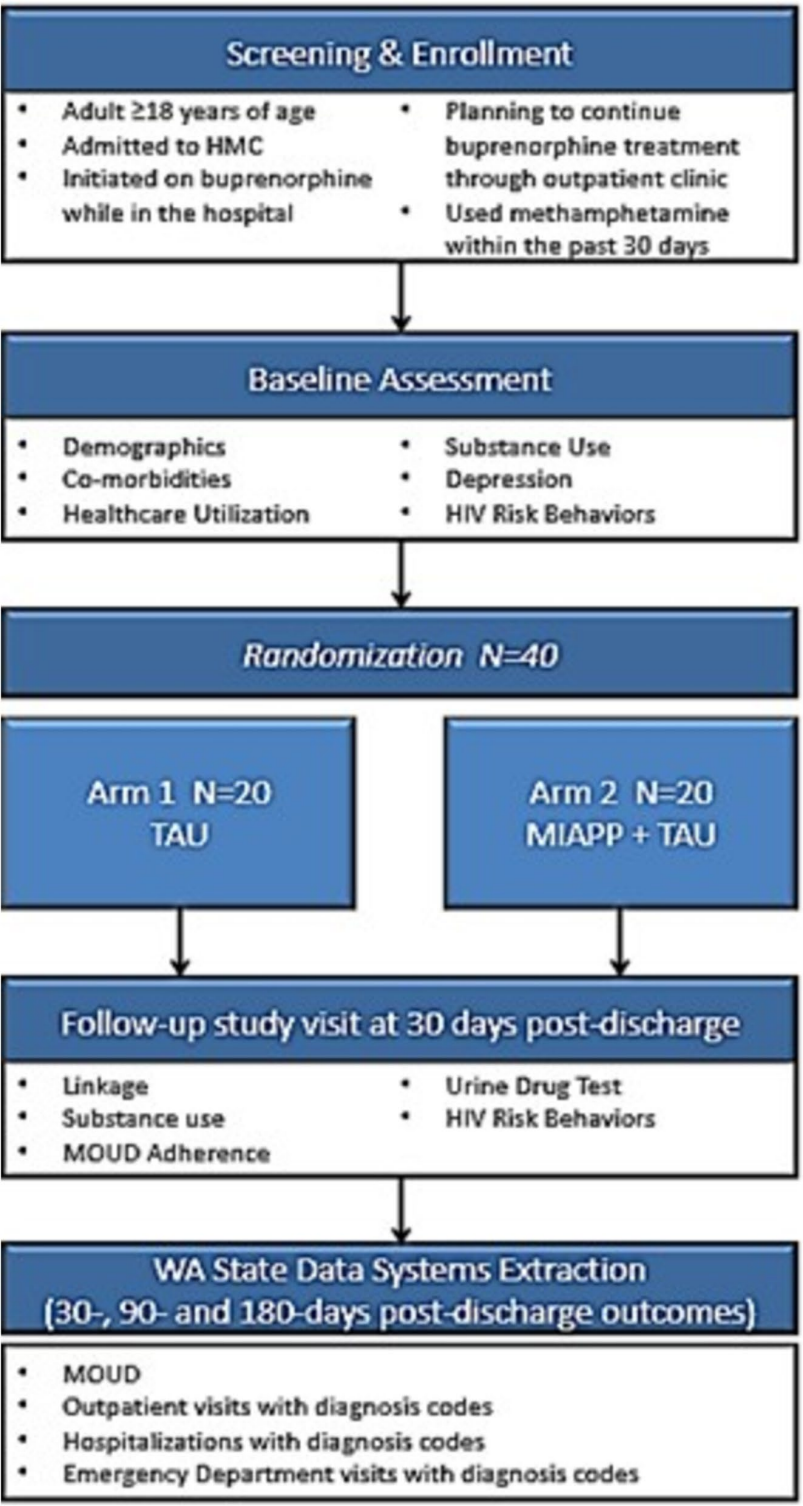
Study flow diagram

To evaluate the feasibility of the intervention and the research procedures, we are conducting a two-arm pilot randomized controlled trial. Forty participants will be assigned to either treatment as usual (N = 20) or treatment as usual + MIAPP (N = 20) using a stratified randomization procedure (Fig. 1).

### Setting

The study takes place at Harborview Medical Center—a large, urban, safety net hospital in Seattle, Washington which has an established Addiction Consult Service comprised of a multidisciplinary team of physicians, nurse practitioners, registered nurses, peer support specialists and administrators who specialize in the treatment of substance use disorders. The Addiction Consult Service is available to all inpatient hospital services (in-person Monday through Friday and via phone or secure messaging through the electronic health record on the weekends) and provides medication management recommendations, brief behavioral counseling, harm reduction counseling, and support around initiating medications for OUD for hospitalized patients and maintaining them post-discharge.

### Study procedures and data collection

Patients who are potentially eligible are referred to the study by Addiction Consult Service team members for screening. The research staff work closely with the Addiction Consult Service team to identify patients to screen: they attend the Addiction Consult Service morning rounds at least weekly and are available throughout the day by phone, email, and through the electronic medical record messaging system. Study flyers are available for research and clinical staff to give to patients. Eligibility criteria are assessed by patient self-report and review of electronic health records and includes: age ≥ 18 years; English speaking; admitted to Harborview Medical Center on any inpatient service; initiated on buprenorphine for OUD while at the hospital or at the time of discharge; planning to continue buprenorphine as an outpatient; and use of methamphetamine within the past 30 days. Participants must be willing to be randomized to video-DOT, willing and able to use a smartphone (which the study can provide if the patient does not have), and willing to work with a Patient Navigator. Participants must be discharged to a setting that allows the use of video-DOT and allows them to attend the follow-up visit 30-days post discharge. Exclusion criteria include cognitive impairment (acute or chronic) resulting in inability to provide informed consent, current incarceration or planned discharge to jail/prison, or exhibiting any behavioral risks per the judgment of the research team.

If the patient is eligible and agrees to participate in the study, the research staff obtains written informed consent, conducts the baseline survey, and randomizes the patient using a REDCap randomization module, typically all within the same day and while the patient is still admitted to Harborview Medical Center. Randomization occurs with permuted block sizes of 4 and is stratified by whether the patient has had buprenorphine treatment in the year prior to this initiation, as prior research shows this treatment history is positively associated with the study primary outcome (linkage to outpatient OUD treatment) [38]. Participants are informed of their randomization assignment by research staff after completion of informed consent and the baseline assessment.

Research assessments occur at baseline and 30 days post-discharge (Table 1). Baseline assessments occur in-person while the patient is hospitalized; follow-up assessments are ideally in-person but can be completed remotely (e.g., by phone). Point-of-care urine drug testing is administered at the 30-day post-discharge visit. Results are recorded for study purposes only and not shared with clinical providers. Participants are reimbursed $60 for the baseline, $60 for the 30-day post-discharge assessment, and $20 for providing a urine sample at the 30-day post-discharge assessment (up to $140 total for research assessments). This reimbursement for study participation is provided to both arms. Data extraction for secondary outcomes (described below) occurs up to 180 days after discharge.

**Table 1:**
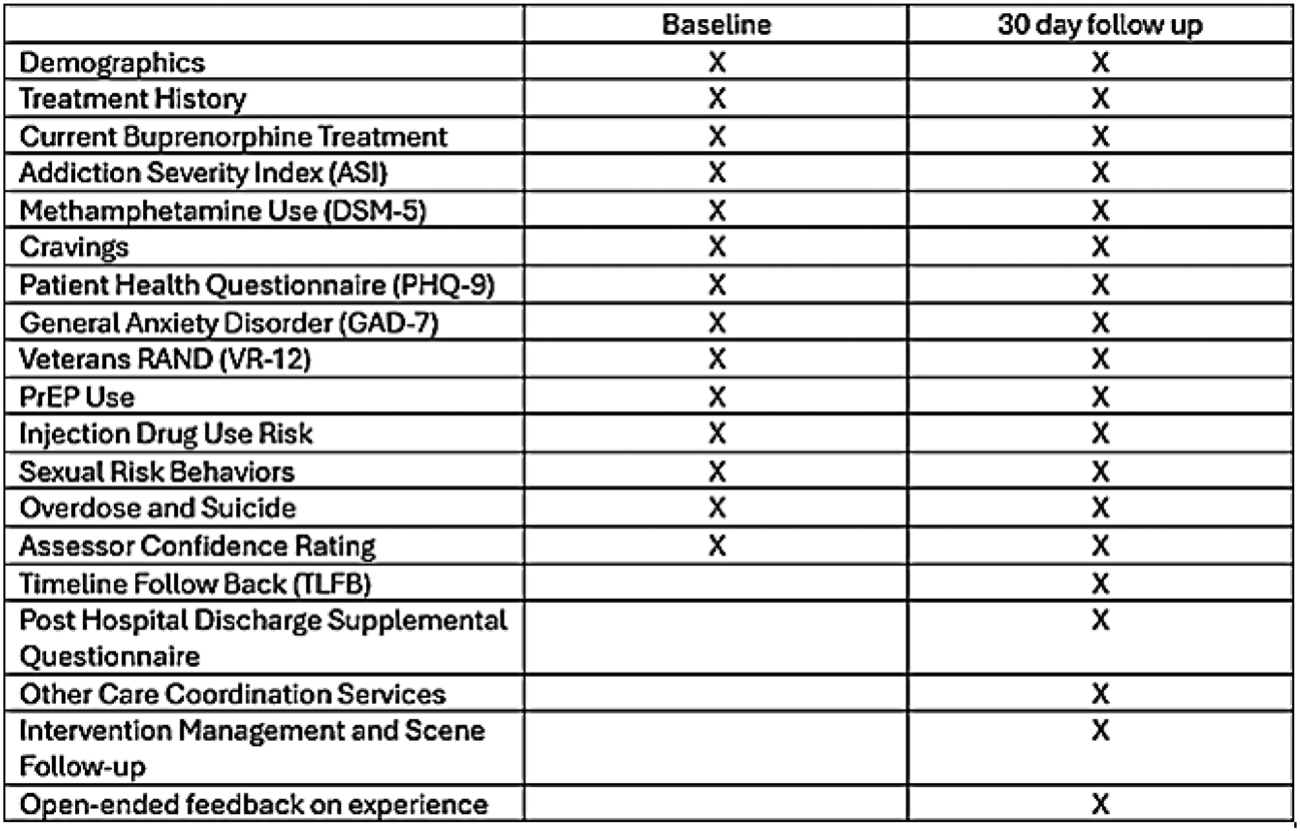
Baseline and 30-day follow-up instruments

### Treatment as usual

Participants randomized to either condition receive treatment as usual from the Addiction Consult Service. These services typically include medication initiation and titration, peer specialist support, and coordination of outpatient follow up. The Addiction Consult Service team routinely sets up the initial outpatient follow up visit and assures that the patient is aware of the plan.

### MIAPP intervention

**Fig. 2:**
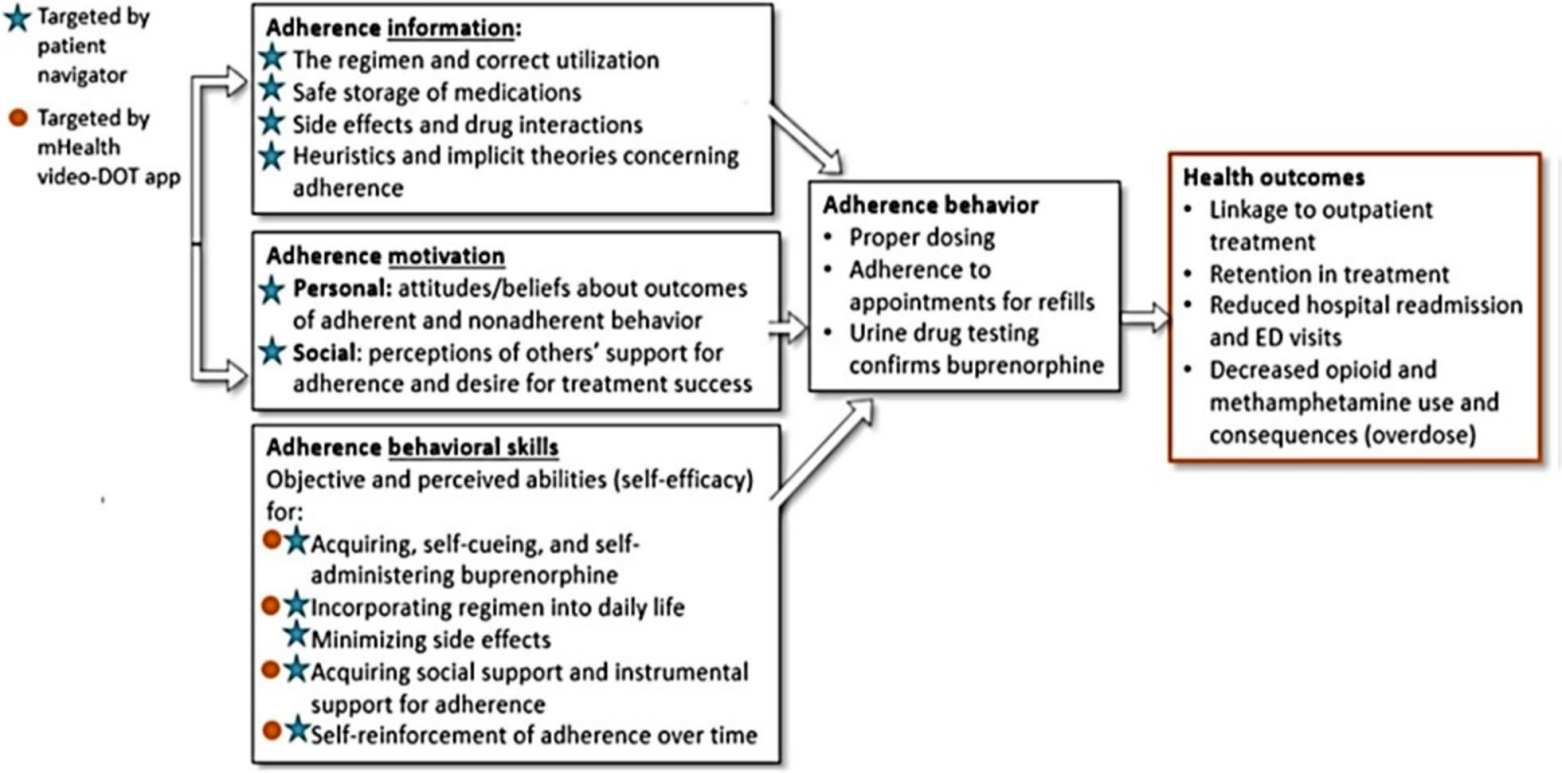
IMB model adapted for buprenorphine adherence

Participants randomized to the MIAPP intervention receive MIAPP + treatment as usual. MIAPP consists of a Patient Navigator with the mHealth adherence application facilitating telehealth visits, two-way chats, video-DOT, and tracking of financial incentives. The components of the intervention are informed by the information-motivation-behavioral skills model [39] (Fig. 2) and are described below.

#### Patient navigation

The Patient Navigator role includes care coordination supported by brief motivational interviewing techniques and focused discussions on buprenorphine medication adherence aimed at enhancing patients’ knowledge, motivation, and behavioral skills for taking their buprenorphine. The initial visit takes place in the hospital and includes introduction, motivational enhancement, care planning, and training on and setup of the mHealth adherence application on a smartphone (either the patient’s smartphone or a study-provided smartphone). If discharge occurs > 3 days after the instruction, then the Patient Navigator performs a “booster” session to review details of using the app just prior to discharge.

The Patient Navigator has scheduled weekly visits with participants and provides services for 30 days post-discharge. For these visits, video encounters are the preferred modality, although telephone calls are also available (e.g., per patient preference or need). Between weekly visits, the navigator communicates via text, telephone, or video exchange through the smartphone app.

The Patient Navigator role is carried out by two hired research coordinators with a bachelor’s and master’s level education. The primary Patient Navigator helped develop the intervention protocol and completed 14 h of training focused on motivational interviewing prior to intervention launch, followed by 2 h review of Motivational Interviewing skill usage during the study with a research team member who is a clinical psychologist. The study team developed guides with checklists for the initial and follow-up visits as well as a care plan template (Appendix 1). The Patient Navigator completed multiple rounds of role plays with these guides and checklists prior to study initiation.

#### mHealth application

The mHealth application was designed by Scene Health and is a digital platform for asynchronous video-DOT. Patients use the HIPAA-compliant, cloud-based mHealth application to securely transfer videos of themselves taking medications, which can be viewed by their Patient Navigator on a Patient Navigator-facing web portal to confirm medication adherence. Earlier versions of the application have been shown to be feasible and acceptable for patients taking buprenorphine [36, 37] and methadone [40, 41].

The application also supports Patient Navigator-patient connections via two-way text messaging and synchronous and asynchronous video communication. Other features include daily medication reminders, a calendar for patients to review their medication adherence, and video messages aimed at providing positive reinforcement upon video submission. Patients are encouraged but not required to share a summary of their adherence with their buprenorphine prescriber. Patients sign a release of information to allow for communication between the Patient Navigator and buprenorphine prescriber in the event the patient navigator should need to communicate important health information to their provider. The application allows patients to list and update their personal recovery goals, access links to websites that offer community support, and resources to help patients log into their electronic health record portals (e.g., to view medical charts and communicate with medical providers).

#### Financial incentives

In addition to motivational enhancement and care coordination provided by the Patient Navigator both in person and through the mHealth app, the MIAPP intervention incorporates financial incentives for medication adherence. In the intervention arm, participants can receive up to $520 in financial incentives through the MIAPP intervention, including $70 for linkage to an outpatient buprenorphine clinic (evidenced by medication refill by outpatient provider) and $15 for each submitted video documenting daily adherence to medication for up to 30 days (Table 2).

**Table 2:** Potential compensation amounts for each treatment arm

	Treatment as usual (both arms)	Intervention financial incentives (MIAPP arm only)
Baseline assessment	$60	$60
End of study assessment	$60	$60
End of study urine drug test	$20	$20
Proof of linkage to outpatient buprenorphine	X	$70
Video to document taking daily buprenorphine	X	$15 per daily video (max $450 over 30 days)
Maximum total compensation	$140	$660

Financial incentive payments are provided as cash through in-person interactions with the research staff who also function as Patient Navigator. Research staff strive to administer rewards as frequently as possible (e.g., weekly) to maximize reinforcement; however, the frequency of reward disbursement is flexible per patient preference and ability.

#### Patient Navigator documentation and auditing

The Patient Navigator keeps a running log of interactions with patients, including the modality (in-person, telehealth, phone, or text), duration, topics discussed, and intervention tasks accomplished. A study clinician/co-investigator reviews the first 10 videos with the Patient Navigator to ensure their competency in approving videos. To ensure intervention fidelity, checklists, logs, and video submissions are audited by research team members every 3 months.

### Outcomes and measures

#### Primary outcome

The primary outcome of interest is successful linkage to an outpatient program that provides medications for OUD within 30 days of hospital discharge, a definition used by prior researchers [42]. This is defined as documentation of an outpatient clinical encounter (either in-person or via telemedicine) where a medication for OUD (either buprenorphine, naltrexone, or methadone) was provided or prescribed. We query participants at the 30-day follow-up visit to determine whether they have successfully engaged in outpatient treatment, which included medications, and we will obtain medical records from the setting they report receiving treatment from to verify. The majority of patients seen by the Addiction Consult Service (~ 75%) have planned follow-up in University of Washington Medicine’s outpatient programs [38]; for these individuals, the primary outcome can be captured by direct review of University of Washington electronic health records. To capture data on participants who follow up outside of the University of Washington, we identify the location where follow-up is planned at the time of study enrollment and complete a “Release of Information” form allowing the research team to obtain records from that location. We will also utilize Medicaid claims data and methadone records from statewide opioid treatment programs through Behavioral Health Data Systems accessed by the Washington Department of Social and Health Services and the Research and Data Analysis Division. Across these data sources, we anticipate this will provide complete data on the primary outcome of treatment linkage for ≥ 95% of patients.

#### Secondary outcomes

1. *Retention on* medications for OUD: This will be measured as the number of days with medication coverage (buprenorphine, naltrexone, or methadone) over the 90 days post-discharge. Data will be extracted by the Research and Data Analysis Division of the Washington State Department of Social and Health Services from statewide Medicaid medication claims data (for buprenorphine and naltrexone) and Behavioral Health Data Systems data (for methadone) as provided by opioid treatment programs.
2. *Hospital readmissions and Emergency Department (ED) visits*: The number of hospital admissions and ED visits within 90 and 180 days of discharge will again be extracted by the Washington Department of Social and Health Services and the Research and Data Analysis Division from statewide Medicaid claims data. We specify 90 days as the main secondary outcome but may also examine up to 180 days.
3. *Past 30-day opioid and methamphetamine use*: This will be defined as the number of days using illicit opioids or methamphetamine in the past 30 days per self-report as collected at the 30-day follow-up interview through the modified Addiction Severity Index, which is a validated measurement tool for assessing substance use [43]. We chose this as a secondary outcome over urine drug testing as we wish to capture reductions in cumulative substance use that may not be detectible through urine drug testing and because results from urine drug tests typically only reflect opioids and other substances used within the past 24–72 h. During the initial months of buprenorphine treatment (i.e., stabilization phase), many/most patients may still use illicit opioids. We will also analyze data on presence of illicit opioids/stimulants in urine drug testing at 30 days, although it is not selected as a secondary outcome for the reasons listed above as well as the higher likelihood that urine drug testing data may be missing (i.e., requires an in-person visit).

#### Other measures

*Demographics*: Age, sex and gender, race, ethnicity, married/partnered status, number of dependents, education, employment status, housing status, and incarceration history will be assessed at baseline by self-report.

*Substance use, HIV risk behaviors, co-morbidities, and healthcare utilization*: Additional variables assessed through questionnaires will include the type of opioid used (e.g. heroin, illicit synthetic opioids and prescription opioids), polysubstance use, injection drug use [44], HIV risk behaviors [45, 46], Charlson Co-Morbidity Index [47], depressive symptoms [48], primary and secondary diagnoses listed on hospital discharge, and history of healthcare utilization in the preceding 90 days, including prior addiction treatment. These measures will be collected at baseline only to characterize the sample, assess randomization equivalence, and control for baseline variables in statistical analyses if needed (e.g., if randomization failure is evidenced by differences between study conditions on baseline measures).

*Adherence*: At the 30-day post-discharge visit we will include measures of self-reported medications for OUD adherence. This will allow us to characterize medication adherence by self-report among the two arms of the pilot randomized controlled trial. Self-reported medications for OUD adherence will be measured in three ways. First, using a visual analog scale from 0 to 100, participants will report the percentage of medications for OUD taken in the past month, as has been used to assess adherence to HIV medication [49]. Second, we will use a validated 3-Item Self-Report Measure for Medication Adherence that was developed for HIV-related and non-HIV-related medications [50]. Finally, we will use a 30-day Timeline Follow-Back method as we have used for prior studies [51].

### Research data management and monitoring

All data from eligibility screenings, research visits, EHR reviews, and adverse events are collected via REDCap [52]. Access to the REDCap servers is provided by the University of Washington’s Institute for Translational Health Sciences. Data is protected by using unique study IDs and stored in password protected computers and programs with only trained research staff having access. Identifiers needed to track participants are kept separate from research data. All videos uploaded by video DOT participants are encrypted and stored in separate site-specific HIPAA compliant web-based Scene Health entities. Only approved research staff members have access to their data. A Data and Safety Monitoring Board will review the safety of participants, collected adverse events and the validity and integrity of the data annually or after half the sample has been recruited (whichever occurs first). The study was approved by the University of Washington and the WA State IRB.

### Statistical analysis plan

Descriptive statistics will be calculated for all baseline variables; variables will be assessed for any differences between the two randomized arms using chi-square tests, t-tests, or the Mann–Whitney U test depending on the distributions. Baseline variables that differ between the two arms and potentially confound tests of intervention effects will be included in analyses as covariates. This study will use an intent-to-treat analysis including all subjects according to their randomized assignment, regardless of their follow-up status and their level of compliance with intervention components. The investigators will provide regular written reports to the Data Safety Monitoring Board including outcome data by the unblinded treatment group.

#### Primary outcome analyses

We hypothesize that participants randomized to MIAPP + treatment as usual compared to treatment as usual alone will be more likely to link to outpatient treatment with medications for OUD within the 30 days of hospital discharge. To evaluate the primary hypothesis, we will use modified Poisson regression analysis [53] with robust standard errors to compare intervention arms on the proportion of participants who link to outpatient treatment with medications for OUD within 30 days of hospital discharge, while potentially controlling for any baseline variables that differ between the two groups as described above if they could confound the hypothesis test. The results of this analysis will be presented as the proportion of patients in each group who link to outpatient treatment and the (adjusted) risk ratio (RR, i.e., ratio of the two proportions) with a 95% confidence interval computed by exponentially transforming the coefficient of the treatment effect in the modified Poisson regression model. We will also report the difference between proportions with a 95% confidence interval, as recommended by CONSORT guidelines [54]. The RR and difference are two different ways of quantifying the intervention effect that have meaningful clinical interpretations. Results from the Poisson regression will be used for primary interpretation and conclusions.

#### Secondary outcome analyses

We hypothesize that participants randomized to MIAPP + treatment as usual compared to treatment as usual alone will (a) have more days of medications for OUD treatment within the 90 days post-discharge (secondary), (b) have lower rates of readmissions to the hospital and emergency department visits within 90 days (exploratory), and c) have fewer days using opioids or methamphetamine 30 days post-hospital discharge (exploratory). Analyses of secondary outcomes will use modified Poisson regression similar to those described above for binary outcomes (readmissions to hospital and emergency department) and linear regression for continuous outcomes (e.g., days of medications for OUD treatment; days of opioid or methamphetamine use).

### Sample size and power

The primary goal of this pilot trial is to develop and pilot test the MIAPP intervention. This pilot work will provide evidence of the feasibility/acceptability of the MIAPP intervention and research procedures that will be used to inform a future, larger study, as assessed by our ability to fully recruit the planned sample and deliver the intervention to the majority of those randomized to the intervention arm. The sample size of 40 for the pilot randomized controlled trial was chosen as we projected we could enroll and complete the study procedures and analyses within the available funding period. Although the sample size was not selected for the purpose of providing a fully-powered study, with our sample size of 40 we are nonetheless powered at 80% to detect some effects if they are large in size. For binary outcomes, power depends on the base rate of the outcome measured, and based on prior studies [38, 55], we anticipate that anywhere from 20 to 50% of participants in treatment as usual successfully linking to outpatient medications for OUD services post-discharge. For binary outcomes, including the primary outcome measure, our pilot study will be powered to detect intervention effect sizes of RR ≥ 1.89, 2.05, 2.40, or 3.10, if linkage rates for treatment as usual are 50%, 40%, 30%, or 20%, respectively. For continuous outcomes (days of buprenorphine coverage, days of opioid or methamphetamine use), we will be powered to detect standardized effect sizes of Cohen’s d ≥ 0.91 with 40 participants.

## Discussion

The MIAPP pilot study will test a novel intervention to improve buprenorphine adherence and linkage to outpatient treatment for hospitalized patients with OUD who also use methamphetamine. While inpatient hospitalizations can be an ideal time to engage with patients and initiate medications for OUD, the transition from inpatient to outpatient care is a vulnerable step in the care cascade where patients may discontinue treatment and become lost to follow-up. The MIAPP intervention is a novel combination of elements including financial incentives, motivational enhancement, and care coordination that may increase the likelihood that patients will continue their buprenorphine and successfully link to outpatient treatment so that they can experience the full benefits of treatment. This initial pilot will be the first opportunity to test the delivery of this new intervention to a sample of patients with OUD and methamphetamine use who are started on buprenorphine. This will allow us to identify unique challenges to enrolling into a clinical study in an inpatient setting and to assess how receptive the target population is to intervention being offered.

In summary, there is an urgent need to improve linkage to and retention in outpatient opioid use treatment, especially for people who use both opioids and methamphetamine. The MIAPP study will evaluate the feasibility of a novel intervention which utilizes the combination of mHealth facilitated incentives and the human connection of a patient navigator to improve transitions from hospital to clinic.

## Supplementary Information

Supplementary Material 1.

## Data Availability

No datasets were generated or analysed during the current study.

## Abbreviations

OUD: Opioid use disorder
MIAPP: mHealth Incentivized Adherence Plus Patient Navigation
mHealth: Mobile health
RR: Rate ratio
