# Supplementary material for: mHealth Incentivized Adherence Plus Patient Navigation (MIAPP): protocol for a pilot randomized controlled trial to improve linkage and retention on buprenorphine for hospitalized patients with methamphetamine use and opioid use disorder
Source: Addict Sci Clin Pract. 2025 Jan 29;20:6. doi: 10.1186/s13722-025-00538-1 (PMC11780921; doi:10.1186/s13722-025-00538-1)
Supplement: Supplementary file 1 — Supplementary Material 1. [file 13722_2025_538_MOESM1_ESM.pdf]

Appendix 01: Care Plan Template

**Patient Study ID:**	**Date:**
**Next Buprenorphine (Suboxone) Appointment:**<br>Time:<br>Date:<br>Place:<br>Transportation:	
**Next talk with the patient navigator:**<br>Date:<br>Time:	
**Key reasons for taking buprenorphine:**	
**Plans for how to get to buprenorphine clinic:**	
**Other goals, thoughts for the week, or future topics; “Is there anything else you are thinking about or that you'd like to add?"**	
**Useful phone numbers:**	
**Patient Navigator name:**<br>**Patient Navigator phone number: 206-496-8064**<br>**Patient Navigator signature:**<br>**Date completed:**	

Appendix 02: First Visit Checklist and Guide

Patient Study ID: ________________

**Tasks**<br>**(Mark when complete)**	**Example Questions/Statements**	**Notes**
**1. Describe the PN's role**<br><br>☐ General Introduction<br><br>**1.2 Agenda**<br><br>• Reasons for taking Bup<br>• Financial Incentives<br>• Scene Onboarding<br>• Bup dose/technique<br>• Transportation<br>• Med Adherence<br>• Care plan	As a patient navigator, there are a few different ways that I can help you with your buprenorphine treatment while you are in the MIAPP program.<br><br>During this visit I hope we can talk through your goals and plans for after you leave the hospital.<br><br>I will stay connected with you after you leave the hospital, and we can communicate using the Scene App.<br><br>Would now be a good time to talk more about these things?	
**2. Motivational enhancement**<br><br>☐ Elicit patient's reasons for taking buprenorphine.<br><br>☐ Reflect or summarize the benefits patient provides	What would you see as benefits to taking buprenorphine after you leave the hospital?<br><br>What impact would buprenorphine have on the way that you feel, if you were to take it in the future?<br><br>(Summarize the desires, reasons, and plans for taking buprenorphine vocalized by patient.)	
**3. Describe and encourage financial incentives**<br><br>☐ Inform patient that MIAPP will provide incentives for linking with the outpatient clinic as well as for daily videos<br><br>☐ Describe the dollar amounts that are provided for each task	Through the MIAPP program, we can offer you additional encouragement to help you take your buprenorphine once you leave the hospital. Specifically, we can give you financial incentives as a reward for taking your buprenorphine after you leave the hospital.<br><br>After you leave the hospital, we will give you $70 if you show us you completed a follow up visit for your buprenorphine.<br><br>In addition, we will give you $15 for every day you send a video showing that you have taken your buprenorphine.<br><br>So, in total, it is possible to earn up to $520 over the 30 days after you leave the hospital.	

☐ Describe how the money will be given to the patient	I will be helping you along the way.<br><br>Sometimes people ask why we're giving people money for taking their medications. So, I like to tell folks why.<br><br>First, we do this because research shows that it works! Financial incentives like these often help people more regularly take medications like buprenorphine.<br><br>But also, we do this to leverage the brain's reward system. You may have heard that opioids and methamphetamine release chemicals in the brain that feel very rewarding – this is why people often go back to using opioids and methamphetamine repeatedly. Receiving money also feels rewarding and releases those same chemicals in the brain – so giving people money for taking buprenorphine helps them keep feeling a similar kind of reward for taking their medications. So, in other words, money can leverage the same reward system that drugs can, so giving people money for taking buprenorphine can help motivate them to take their medication. How does this sound to you so far?<br><br>What questions do you have?	
**4. Scene app onboarding and training**<br><br>☐ Explain how the Scene app will be used for documenting medication adherence<br><br>☐ Set up Scene App on patient's phone<br><br>☐ Complete Scene training manual	We'll use a smartphone app to help you get those financial incentives. The app is called Scene. You'd use this app to submit videos of yourself taking buprenorphine every day.<br><br>We'll also use the app to communicate once a week after you leave the hospital.<br><br>Fortunately, we've found that most patients are able to learn how to use it fairly quick.<br><br>Would it be alright with you if we got the app set up on your phone now?<br><br>[Refer to the Scene App MOP]<br><br>You'll need to make sure the video records for at least 3 minutes after the buprenorphine is under your tongue. This way, we'll know that you're taking it correctly, because the medication needs to dissolve in your mouth instead of being swallowed.	

	Please continue to take your medication as prescribed in any circumstance where you are unable to upload the video for any reason.<br><br>You can also reach out to me for help if you run into any technical difficulties or need help using the app.	
**5. Review buprenorphine dosing and technique**<br><br>☐ Patient verbalizes dosing schedule<br><br>☐ Provide medication taking techniques<br><br>☐ Provide Bup taking tip sheet	Have you taken buprenorphine in the past, if so, can you tell me about that experience?<br><br>Are you able to describe for me when during the day you will take the buprenorphine, and how much you will take?<br><br>Have you had any trouble taking the buprenorphine while you have been here in the hospital?<br><br>The outpatient nurses in our buprenorphine program have come up with some tips on taking buprenorphine that they share with patients, would it be ok if I shared those with you?	
**6. Assess transportation issues**<br><br>☐ Establish how patient will get to first buprenorphine appointment<br><br>☐ Troubleshoot transportation barriers.	Do you know yet what clinic you'll be getting your buprenorphine from after you leave the hospital?<br><br>How far away is that from where you live?<br><br>How do you plan on getting to that clinic? For example, will you be walking, taking public transportation, getting a ride from someone, or something else?<br><br>Do you have any backup options for getting there?<br><br>What other challenges do you think could stop you from getting to your appointment?	
**7. Assess and troubleshoot potential barriers to medication adherence**<br><br>☐ Acknowledge that medication adherence is difficult for many people.	A lot of people have difficulty taking medications consistently every day. If you find it difficult to get into the habit of taking it every day, that's okay! It happens, and it's hard for a lot of people.<br><br>Have you ever taken buprenorphine before, outside of the hospital? How'd that go, and what challenges did you have with taking it consistently?<br><br>Have you used any strategies in the past to help you take other medications every day?	

☐ Elicit potential barriers to taking buprenorphine<br><br>☐ Suggest strategies to overcome barriers	Some people find that setting an alarm can be a helpful reminder to take your medication. Some people use the buddy system by having a friend or family member check in with them every day to make sure they've taken their medication. Do any of these ideas stick out to you?	
**8. Develop a care plan and go over future appointments**<br><br>☐ Schedule next meeting<br><br>☐ Compete Care Plan Document	Would it be okay to schedule our next meeting now and fill out this care plan, we can do it together.<br><br>You can take this paper to help remember when and where your follow-up buprenorphine appointment will be.<br><br>You and I will also have a phone-based check-in on [day, time]. We will do this by video through your phone, or we can do it through a regular phone call if video doesn't work.<br><br>During that call, I'll check in with you about how you're doing. During that call, I can also help you with problems you might run into with taking your buprenorphine. I can also help you make sure that you're getting your financial rewards.	

Additional Notes:

Patient Navigator name: ________________

Patient Navigator signature: ________________

Date completed: ________________

Appendix 04: Follow Up Visit Checklist and Guide

Pre-Visit Checklist:

☐ Have a copy of the patient's Care Plan.

☐ Review Scene app to approve any outstanding videos or patient messages.

☐ Calculate financial rewards patient has received and amount of rewards still eligible to receive.

- Rewards received to date: ___________
- Rewards still eligible to receive: ___________

Follow up Visit Checklist:

Patient Study ID: _____________

Follow up Visit Number (circle): 1 2 3 4

Date: ___________

**Tasks** (Mark when complete)	**Strategies**	**Notes**
☐ Review the summary calendar<br>☐ Review compensation received<br>☐ Review ups and downs from the week<br>☐ Review buprenorphine appointments<br>☐ Assess potential barriers to attending upcoming appointments	Open ended questions<br><br>View the calendar in the Scene app together.<br><br>How was the past week?<br><br>What went well?<br><br>What was hard?<br><br>I know there are a lot of different reasons someone can miss their appointment. I'm curious what got in the way for you, specifically, for the appointment on [date]?	
**Motivation**<br><br>☐ Use motivational enhancement techniques to ask about the benefits of buprenorphine (if taking), or anticipated benefits (if not taking).	How are you feeling about buprenorphine?<br><br>What are some pros and cons to taking it?	

**Coaching**<br>☐ Discuss adherence strategies	How do you remember to take your buprenorphine?<br><br>Are there any tips or tricks you would recommend to others?	
**Goal setting**<br>☐ Revisit overall goals<br>☐ Emphasize patient strengths and accomplishments		
**Follow up plans**<br>☐ Review care plan<br>☐ Schedule next visit<br>Date of visit:	Are there any goals for this week?<br><br>Are there any phone calls you need help with?<br><br>Let's look at the calendar and find a good time to meet again	

Additional Notes:

Patient Navigator name: ________________

Patient Navigator signature: ________________

Date completed: _______________
